# Comparison of micro-CT imaging and histology for approximal caries detection

**DOI:** 10.1038/s41598-017-06735-6

**Published:** 2017-07-27

**Authors:** C. Boca, B. Truyen, L. Henin, A. G. Schulte, V. Stachniss, N. De Clerck, J. Cornelis, P. Bottenberg

**Affiliations:** 1 Department of Electronics and Informatics – ETRO Vrije Universiteit Brussel, Brussels, Belgium; 2 EXIA, Brussels, Belgium; 3 Oral Health Research Group, Vrije Universiteit Brussel, Brussels, Belgium; 4 Department of Special Care Dentistry, Universität Witten/Herdecke, Witten, Germany; 5 Department of Conservative Dentistry, Universität Marburg, Marburg, Germany; 6 Microtomography Research Group, Department of Biomedical Sciences University Antwerp, Antwerp, Belgium

## Abstract

Histological sectioning is a generally accepted *in vitro* validation method for caries detection techniques. However, it requires cumbersome sample preparation and induces irreversible sample destruction. Micro-Computer Tomography (micro-CT) allows non-destructive imaging of tooth structure. The aim of this study was to compare the performance of histological sectioning and micro-CT imaging in detecting approximal carious lesions. Unlike previous studies, evaluation is objectified by comparing visual appearance of exactly corresponding anatomical regions. Sixty extracted human teeth were scanned with a desktop micro CT system. Axial histological slices were prepared and photographed. Sample preparation, combined with dedicated image processing, ensured selection of identical anatomical regions on radiographic and histological images. Evaluation of the presence and extent of carious lesions was performed by four dentists using custom-designed software. Each section was scored independently (histo or micro CT). Scores of approximal surfaces were retained for further analysis. Spearman’s correlation coefficients (0.738 to 0.829, p < 0.0001) showed a good agreement between signs of carious lesions in the identical region obtained with both methods. Bland-Altman plots showed that 90.76% of the data points were within the limits of agreement. Micro-CT imaging was shown to provide an interesting alternative to histological sectioning as detection method for carious lesions.

## Introduction

Detection of carious lesions is not straightforward, especially if the lesion is obscured by surrounding healthy tooth tissue^1, 2^. In order to complement the conventional visual detection^3^, new techniques are being developed^4^. In order to validate such methods, a systematic comparison with a universally accepted measure is required.

Histological sectioning, be it hemisection or serial sections, is widely used as an *in vitro* validation method for caries detection. However, histological preparation is a cumbersome and labour intensive technique which leads to irreversible sample loss. Moreover, samples can be damaged by fracture or chipping of dental hard tissues, while irreversible destruction or obfuscation of small lesions can be incurred by the saw cut.

Furthermore, histological sections of teeth affected with caries preferably show changes in translucency and colour but do not reveal actual mineral loss^1^. This requires trained and experienced observers to reach consistency in determining the delimitation of a carious lesion.

To date, there is no absolute consensus about the delimitation of carious lesions in histology (Bottenberg and Schulte, ORCA conference abstract nr. 37, Caries Res 2011, 45: 189). The fact that some teams apply additional staining^5^ or microhardness measurements^6^ to determine lesion extent demonstrates that judging the limits of a carious lesion with the aid of optical microscopy presents a challenge when using histology as a reference standard.

Therefore, a non-destructive, precise, and reproducible validation technique would provide a highly viable alternative to histology. Among radiographic imaging techniques, micro Computer Tomography (micro-CT) has the potential to become a promising alternative to histological sectioning as validation method in caries research. Broadly speaking, micro-CT is a miniaturized version of the well-known method of computer axial tomography used in medical diagnosis, but operating at a substantially higher resolution in the order of micrometers^7, 8^. The accompanying high radiation dose currently restricts its use to laboratory studies.

Micro-CT imaging allows the extraction of cross-sectional slices, which can be re-assembled into three-dimensional images using further computer processing. By imaging the degree of mineral loss, micro-CT may permit a more objective determination of the actual extent of the caries lesion than does the interpretation of colour changes on histologic slices as an indirect indicator of demineralization^1^.

Comparison of histology and micro-CT in assessing caries lesion depth has been reported before. Both methods were indirectly compared^9^ using clinical detection methods (radiography and visual examination). This approach could have led to possibly incorrect inferences due to the well-documented limitations in sensitivity of the clinical methods. A direct comparison of micro-CT and histology, conducted on a low number of samples instead, was described by Mitropoulos *et al*.^10^.

Common to both studies is that correspondence in lesion depth was assessed by visually comparing selected histological sections against the full three dimensional stack of micro-CT images. As human intervention is inherently fraught with perception and interpretation errors, and parts of the lesion may have been destroyed by sectioning, a match between the supposedly deepest histological section with a micro-CT section representing the true depth and the anatomical correspondence of the lesion as assessed by both modalities could not be guaranteed. To avoid this disadvantage, it would be of great value to develop an experimental technique that would allow to compare the histological section with an exactly corresponding section from the micro-CT image stack. Another advantage of such a method would be to preclude deviations of the histological and radiographic sectioning plane because by this parallax errors in the assessment of the lesion depth could be avoided.

Such a technical approach was used successfully in a pilot study including 11 teeth^11^. Subsequently, this working group was able to improve the procedure and was able to obtain a better photographic representation of the histological sections.

The aim of the present study is to compare the performance of histology and micro-CT in assessing the extent of approximal caries lesions, based on the visual appearance of corresponding axial sections.

As working hypothesis of this study, it is assumed that micro-CT imaging and histology produced equivalent categorical scores of approximal carious lesions.

## Materials and Methods

The study material consisted of 75 anonymously collected extracted human premolars and molars, stored in a moist environment, and carefully selected using visual inspection to encompass different lesion depths about 20 lesions for each of the 4 lesion depth categories^12^. The non-carious surfaces were used as control. Selection of the teeth was performed by a team member not involved in the later scoring.

Teeth which had been damaged by the caries process so extensively that cavity walls were lacking as well as teeth with any sign of a restoration were excluded. Our team has obtained the permission of the ethical committee of Vrije Universiteit Brussel (VUB) to use extracted human teeth for scientific studies in cariology (ref. B14320096266).

### Micro-CT imaging

A bench-top system, SkyScan-1076 (SkyScan, Bruker, Kontich, Belgium), based on a combination of X-ray projection microscopy and tomographic data reconstruction was used to acquire images of the selected teeth. In this system, an air-cooled X-ray point source (energy range 20–100 keV, focal spot size 5 μm) illuminates the object with a divergent beam, and magnified shadow pictures are detected by a 2D X-ray CCD camera (2.3 × 4 k).

The scans were performed at 100 kV, 100 μA. Scans were isotropic and a voxel size of 18 × 18 × 18 μm was chosen, resulting into 293 two-dimensional projections corresponding to a 180° rotation of the sample with rotation steps of 0.7°. An aluminium filter of 1 mm that attenuates low energy X-rays was used during the acquisition process to reduce the beam-hardening effects.

Reconstruction was performed with the software interface (Nrecon) which had been provided by the manufacturer of the scanner (Bruker SkyScan, Kontich, Belgium) by using a modified Feldkamp cone-beam algorithm^13^, with optimal contrast limits, ring artefact reduction and beam-hardening correction^14^.

**Figure 1:**
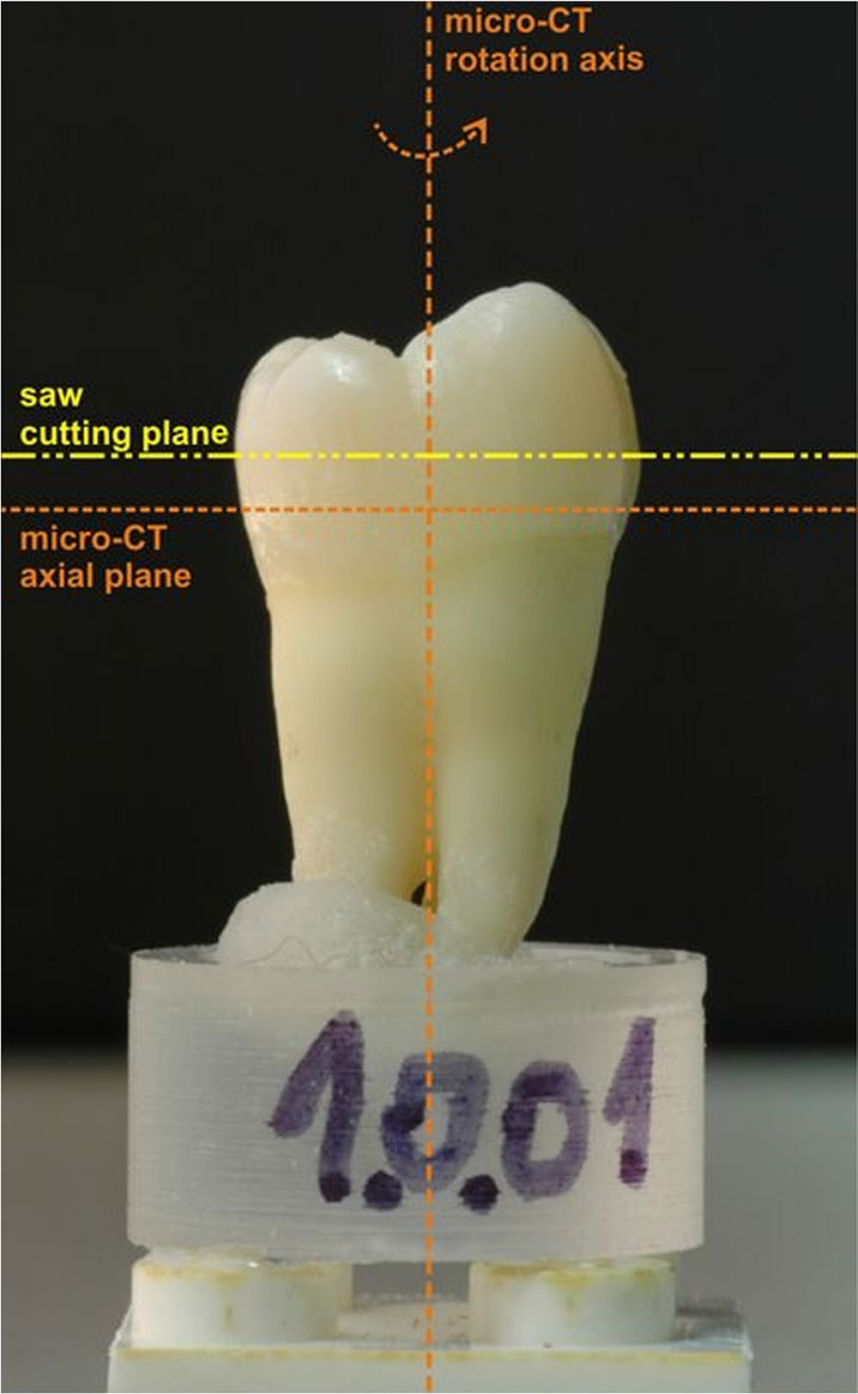
Sample tooth mounted on the custom designed holder.

All teeth were individually mounted on a specially designed support (Fig. 1) consisting of a plastic disc fixed to a Lego® block, to ensure identical slice orientation during micro-CT imaging and histological preparation. The support, on which the tooth was mounted in vertical position, was used in combination with a modified scanner bed with a Lego® baseplate oriented perpendicular to the scan axis. Another Lego® base plate was mounted in the saw, parallel to the cutting blade. This mounting method not only ensured accurate positioning of the sample both during scanning and through the sectioning process but also guaranteed that the transversal planes of the microtomograph and the cutting planes of the histological samples were parallel. Thus the process of matching corresponding slices required retrieval of the slice at the correct height in the stack of images and appropriate scaling, translation, rotation as described below in the section describing the matching procedure.

### Histological evaluation

After obtaining micro-CT images, all teeth were dehydrated and embedded in self-curing acrylic resin and fixed in a microtome with a 300 micrometer thick diamond-coated saw blade (Buehler, Lake Bluff, USA). The embedding guaranteed that sectioning planes of the microtomograph and of the histological samples were parallel. Tooth material was removed by successive grinding in 400 micrometer increments. The sectioning plane was oriented perpendicular to the tooth long axis. Each cut surface was photographed with reflected light using a digital camera (Nikon) equipped with a macro lens. The exact magnification was determined from the image of a calibrated grid (10 lines/mm), placed on the histological sample at an identical focal distance and exhibiting the same magnification. In five teeth the lesion area was damaged during sectioning, 10 others yielded unusable images due to technical problems with the camera. From the remaining 60 teeth, a total of 73 histological sections with 146 approximal surfaces either sound or encompassing different degrees of proximal carious lesions were retained for examination (1 or 2 sections per tooth depending on the size and position of the lesion).

### Micro-CT and histological section matching procedure

One team member not involved in the scoring procedure selected the histological slides showing the largest extension of the lesion. In case that both approximal surfaces presented a lesion, two sections per tooth could be selected. In order to evaluate the micro-CT images against the histological slices, a correspondence between the images had to be established to allow the observers to examine the same site of the lesion. The image sets used in this study were matched using an automated, computer-assisted procedure. Because the axial planes of the micro-CT scan were parallel with the cutting plane of the saw, finding a corresponding image in the CT stack for a given histological slice required finding the image along the Z-axis.

First, dimensional standardization (equal magnification) was performed using the information obtained from the optical measuring grid of the histological images and the voxel size from the image log of the micro-CT apparatus. Images were resized accordingly and cropped in order to reduce the amount of useless information.

In order to achieve selection of corresponding sections, dedicated software was developed to extract the contours of enamel, dentine and pulp in either micro-CT or histological images by thresholding. Slides with corresponding contour lengths were selected. An operator verified this visually and put the selected group of micro-CT slices through a second computer program that chose the corresponding contours by a fully automatic pixel-based registration method, based on concept of mutual information^15^.

### Scoring of the histological and micro-CT images

Before the actual scoring, 15 training slides were shown to the panel and discussed in order to apply a uniform way of scoring. Scoring of the histological and micro-CT sections was performed on a 21″ high-quality computer monitor (Sony, Tokyo, Japan). Proprietary designed software was developed as graphic user interface to perform the scoring. In order to avoid bias, slices were presented to the observers in a randomized order. In order to prevent bias, micro-CT images or histological sections were scored in two separated sessions. Observers had the possibility to enlarge the section and to view micro-CT images in inverted black-and-white modus. The selected images were divided in four quadrants in order to ensure the correct assignment of lesion to the four surfaces of the section. Per image quadrant, a categorical score could be ticked and confirmed by the observer before the next slice was presented. Each surface was scored by four observers independently who had to attribute one out of five categories proposed by Downer^12^:

1. No enamel demineralization or a narrow surface zone of opacity
2. Enamel demineralization limited to the outer 50% of the enamel layer
3. Demineralization involving the inner 50% of the enamel up to the dentino-enamel junction
4. Demineralization involving the outer 50% of the dentine
5. Demineralization involving the inner 50% of the dentine

The sections were scored two times with a 3 months interval between scorings. Only the scores of the approximal surfaces were retained for further analysis. Lesion outline was defined as follows: in histology, the limit of a carious lesion in enamel was considered as the limit of a discoloured area in enamel. In dentine, the lesion was defined as the border between the discoloured zone and the transparent zone^16, 17^. In micro-CT images, the lesion was defined as the transition between light grey values due to demineralization and the darker grey levels of the surrounding healthy tissues (or vice-versa in case an inverted grey level image was chosen by the observer). In both viewing modalities, the shape of a carious lesion was considered to differentiate it from artefacts or non-carious defects.

### Statistical processing

The correlation between the categorical scores of the histological and micro-CT images was determined by Spearman’s rank correlation. Cohen’s kappa was used to measure the inter- and intra-observer agreement for each method. Using a student’s t-test (based on output kappa ± S.E., n = 60 samples, 4 observers) kappa values for both methods were compared. Inter-observer agreement was calculated with Fleiss’ kappa statistics. The difference of distribution of surfaces with different lesion categories was evaluated by means of a χ^2^-test. Statistical calculations were performed using the IBM SPSS Statistics 20 (IBM Corporation, Somers, NY, USA) software package. Bland-Altman plots are used to analyse agreement of two methods by plotting the difference between the values obtained using these methods against the average of the obtained values for each sample. Bland-Altman plots^18^ were generated in Matlab R2010b (The Mathworks, Natick, MA, USA). The agreement interval about the mean difference was chosen as ± 1.96 times the standard deviation.

### Ethical approval

This article does not contain any studies with human participants or animals performed by any of the authors. The use of collected extracted human teeth was approved by the ethical committee of the Medical Faculty/University Hospital VUB (ref. B14320096266). For this type of study, formal consent was not required.

## Results

A total of 146 surfaces were scored by each observer for each technique in duplicate. Out of the surfaces about one third was scored as sound with histology and nearly half with micro-CT. Cross-tabulations for micro-CT and histology per observer and for all surfaces are shown in Tables 1 and 2. Both methods showed comparable results for carious lesions in enamel and in outer dentine (χ^2^, p = 1.0). However, if data were dichotomized into sound (category 1) vs. carious (lesion categories 2–5), χ^2^ was significant (p < 0.001) as well as for the dichotomization between lesion categories 2–4 vs. 4 (χ^2^, p < 0.01). Thus, more surfaces were categorized as having deep dentine caries in histology than with micro-CT and less surfaces were scored as sound in histological slides compared to micro-CT (Table 3).

**Table 1:** Number of surfaces and their scores in 5 categories, obtained by scoring micro-CT (μCT) and histological images (Histo).

Lesion category	Observer								Total	
	1		2		3		4		Histo	μCT
	Histo	μCT	Histo	μCT	Histo	μCT	Histo	μCT		
Sound (0)	56	71	51	69	55	65	51	68	213 (36.5%)	273 (46.7%)
Outer enamel (1)	14	16	29	21	21	21	20	13	84 (14.4%)	71 (12.2%)
Inner enamel (2)	17	15	10	13	13	16	17	21	57 (9.8%)	65 (11.1%)
Outer dentine (3)	38	33	34	31	34	34	32	31	138 (23.7%)	129 (22.1%)
Inner dentine (4)	21	11	22	12	23	10	26	13	92 (15.6%)	46 (7.9%)
Total	146	146	146	146	146	146	146	146	584	584

Number of surfaces and their scores in 5 categories, obtained by viewing microCT and histological images.

**Table 2:** Crosstabulation of number of surfaces scored by observing histological (columns) or micro-CT (rows) slices, per category. Corresponding categories are given in bold figures.

Micro-CT→	Sound	Outer enamel	Inner enamel	Outer dentine	Inner dentine	Σ
Histo↓						
Sound	**196**	10	3	3	1	213
Outer enamel	39	**34**	10	1	0	84
Inner enamel	18	16	**18**	5	0	57
Outer dentine	14	11	27	**83**	3	138
Inner dentine	6	0	7	37	**42**	92
Σ	273	71	65	129	46	584

Cross tabulation of number of surfaces scored by observing histological or micro-CT slices, per category. Corresponding categories are given in bold figures.

**Table 3:** Dichotomizing surfaces between lesion categories detected with histology (Histo) or micro-CT (μCT) as given below yielded a significant χ2 test.

Categories:	Histo (n, %)	μCT (n, %)
Sound (0)	213 (36%)	273 (47%)
Carious (1–4)	371 (64%)	311 (53%)
Significance χ2-test:	p = 0.0005	
Carious lesions (1–3)	279 (75%)	265 (85%)
Inner dentine lesions (4)	92 (25%)	46 (15%)
Significance χ2-test	p = 0.015	

The overall correlation between micro-CT and histology was very high: 0.80 (p < 0.0001) with individual observer’s Spearman correlation coefficients ranging from 0.79 to 0.83. Reproducibility for each method was evaluated using Cohen’s kappa. Intra-examiner agreement was substantial for both histology and micro-CT (Table 4). Applying the t-test for intra-examiner kappa ± SE), the difference between histology and micro-CT was significant (p = 0.005). Inter-observer agreement for histology had an inter-rater kappa coefficient of 0.86. Micro-CT had an inter-rater kappa coefficient of 0.88 (Table 4), p < 0.05.

**Table 4:** Agreement (as expressed by intra- and inter-rater Kappa) for histology and micro-CT.

Observer	Histo (kappa ± SE)	Micro-CT (kappa ± SE)
1 (intra)	0.64 ± 0.05	0.73 ± 0.05
2 (intra)	0.74 ± 0.04	0.81 ± 0.04
3 (intra)	0.75 ± 0.04	0.80 ± 0.04
4 (intra)	0.71 ± 0.02	0.83 ± 0.04
**All (intra)**	**0.71 ± 0.02**	**0.79 ± 0.02**
**All (inter)**	**0.86 ± 0.01**	**0.88 ± 0.01**

**Figure 2:**
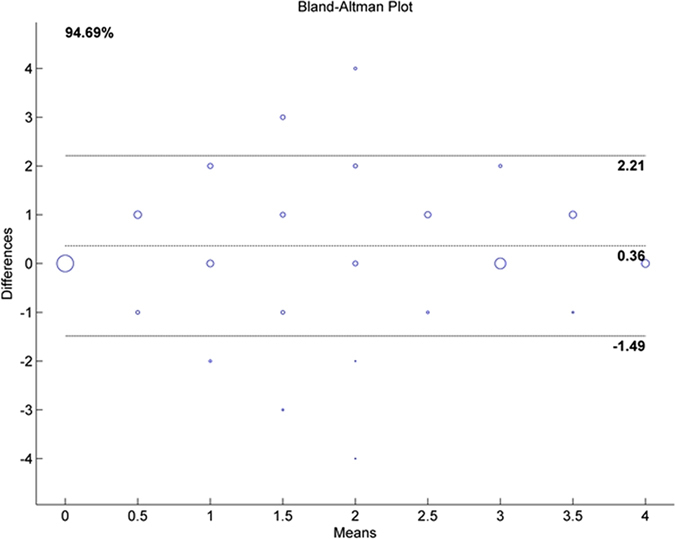
Bland-Altman plot for all slices. Bubble size is proportional to the number of samples.

After calculating the difference between the histological and microtomographic scores, 64% of the surfaces showed no difference. Second and third in frequency was a number of surfaces with a positive difference (+1, 20%, +2, 6%), indicating a higher score for histology. A higher score for micro-CT (−1) was obtained in 5% of the surfaces. This was confirmed in the Bland-Altman plot (Fig. 2) with a mean difference (score histology-score micro-CT) between the individual scores of + 0.36 ± 0.94, indicating a general higher score of histology. However, 94.7% of all data points were within the limits of agreement.

## Discussion

The sample preparation procedure, with the custom designed holders, and the automatic selection and matching of the micro-CT sections with the histological sections allowed precise comparison of identically located images. In similar studies, comparison was made using the entire stack of micro-CT images^10^. Soviero *et al*.^9^, Özkan *et al*.^19^ and Taylor *et al*.^20^ manually selected the images to be compared. Furthermore, in contrast to the above-mentioned studies, the sectioning plane differed by 90 degrees, being mesio-distal instead of occluso-apical. Histological sections do not necessarily contain the deepest point in the lesion. Nevertheless, in the cited studies, for a given lesion, the histological slice was compared against the deepest point of the lesion observed on the Micro-CT. Thus an underestimation of the depth of the lesion on the histological slice was possible. Furthermore, in these studies, a lower number of surfaces, or observers and a possible imbalance of caries-free surfaces with other lesion types could have influenced the statistical outcome. The slice to slice comparison used in our study avoided this bias and allows comparing lesions at exactly corresponding sites.

In principle, lesion assessment can be done quantitatively by measuring the lesion depth or qualitatively by assigning the lesion to categories as was proposed by Downer^12^, Ekstrand *et al*.^21^ and the ICDAS method^22^. The latter approach was applied in the present study because the authors realised that in most studies involving histology as reference standard for caries detection the carious lesions were categorized in this way.

**Figure 3:**
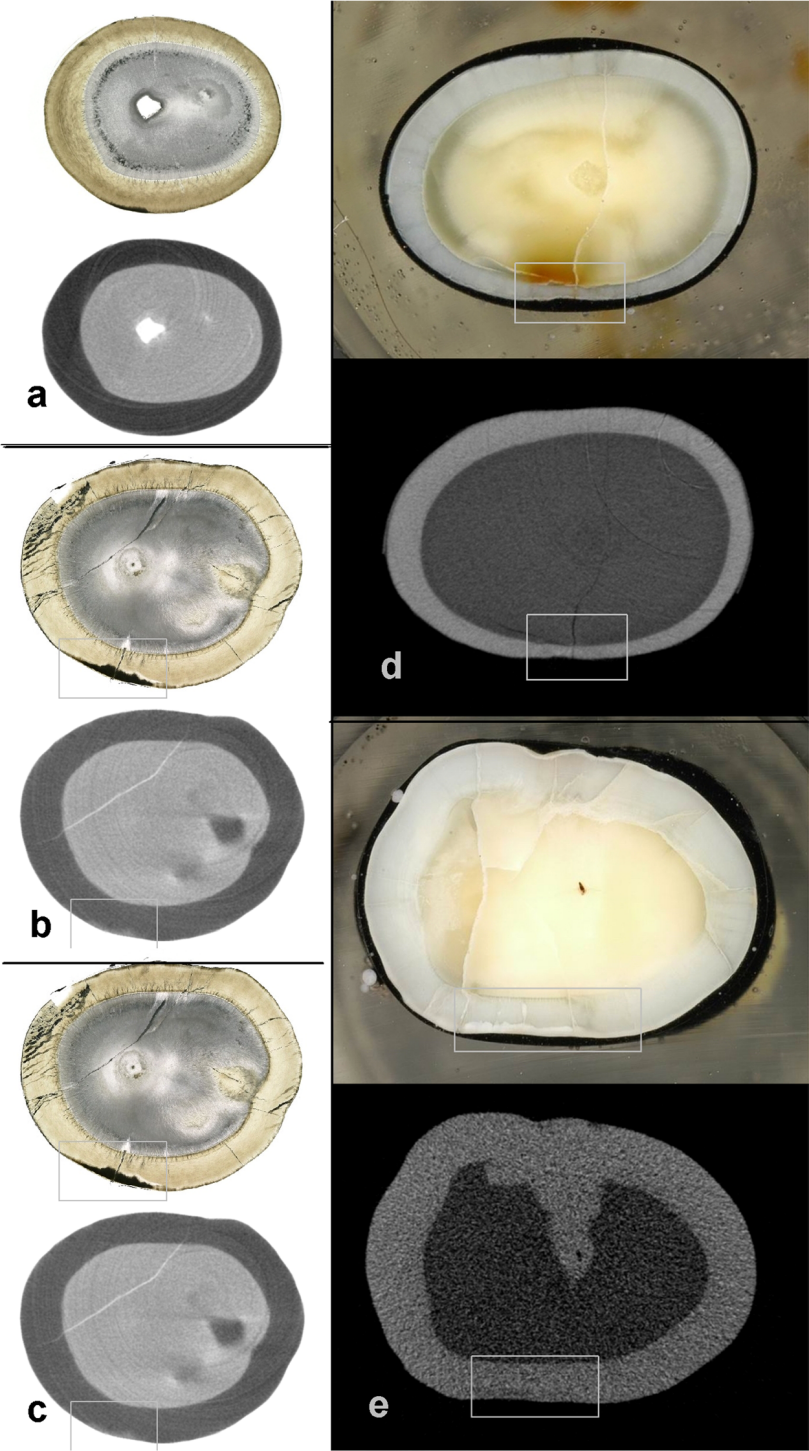
Exemples of histological or micro-CT images showing agreement or disagreement between histology or micro-CT scored unanimously by all 4 observers.. Agreement, (a: sound, b: enamel caries, c: dentine caries), disagreement is shown in image d: higher score in histology than in micro-CT and e: higher score in micro-CT compared to histology.

Using assessment of histological samples as reference technique allowed us to calculate a sensitivity of 0.72 and specificity of 0.97 at a cut-off at lesion category 1 for the assessment of micro-CT samples. Using lesion category 3 as cut-off yielded nearly the same values. Even if the concept sensitivity and specificity does not fully apply in the present experiment, it confirms that both methods agree better on sound surfaces than on carious ones (Fig. 3). Due to the successive grinding technique an absolute gold standard in the form of microradiography^23^ could not be achieved. Despite the comparison at identical anatomical location, a perfect correlation could not be reached. With regard to the superficial enamel lesions (E1) and the deepest dentinal lesions (D2), micro CT and histology performed in a significantly different way. However, micro-CT reached higher inter- and intra-rater agreement than histology.

The observed discrepancy in detection of small enamel lesions cannot be explained by a possible lower detection capacity or resolution of micro-CT, since Hamba *et al*.^23^ showed that micro-CT was equivalent to microradiography, a technique widely accepted as reference for the detection of early enamel lesions. Therefore, we explain the observed discrepancy rather by the fact that some of the small lesions may have been remineralized but behaved optically in a different way in histology without showing a difference in mineralization in micro-CT.

The discrepancy observed in deeper dentinal lesions can be attributed to the fact that a colour change in dentine may not only be caused by the carious process but also by microbial metabolites or external stains such as coffee or tobacco without being related to demineralization or bacterial infection^1^.

Further discrepancies can be explained by factors associated with the different capabilities of human observers. Apart from a different vision capability to distinguish between different grades of colour or grey-level the observers may have had different views about the extent of a lesion. Without a measuring device, observers judging the depth of a lesion can attribute it to a different lesion category. In several studies, inter-observer agreement (kappa coefficient) of less than 1.0 was mentioned when a histological reference for caries detection was performed^17, 24^. In the future, using an appropriate standardization and segmentation technique, an automated delimitation of the carious lesion with micro-CT^25^ may overcome this problem.

It can be speculated that micro-CT imaging may be subject to specific limitations caused by artefacts in the reconstructed images^26^ such as beam-hardening, which is caused by polychromatic radiation sources used in currently available desktop micro-CT scanners. Higher X-ray energies of the polychromatic spectrum are less attenuated while the lower energies are easily absorbed, which results in a cupping artefact (increased density) at the tooth’s edge. However, correction of beam-hardening artefacts can be performed by linearizing the relationship between attenuation and material thickness based on a calibration curve. This calibration can be obtained using a wedge-shaped phantom from a material with a similar composition^27^ or from an initial reconstruction in which the scanned object serves as internal standard^28^. In order to estimate the effect of beam hardening correction we compared a subset of 15 samples which were reconstructed with and without beam hardening correction, as provided by the reconstruction software Nrecon (SkyScan, Bruker, Kontich, Belgium), by assessing the correlation between histology and micro-CT. When no correction was applied, Spearman’s correlation coefficient between micro-CT and histology decreased from 0.719 (p < 0.0001) to 0.357 (p < 0.0001). This leads to the conclusion that improper artefact reduction can influence the performance of micro-CT imaging. In a future study the quantitative differences between various correction techniques and their effect on the performance of the validation method will be investigated. Beam hardening by the aluminium filter did probably reduce some of the possible artefacts (which could first of all not be tried experimentally because it would interfere with the function of the device) but there is also a beam hardening effect of the sample itself^29^. A tooth being a rather large size solid radiopaque object inevitably absorbs radiation in a different way than bone or mollusc shells containing more air spaces^30^.

It is concluded that, even in view of its specific limitations, micro-CT imaging of teeth represents an interesting alternative to histology as detection method for carious lesions. In addition, micro-CT imaging of teeth has the potential to be accepted as a further validation method in caries research, provided that our results are confirmed by other author groups. A pre-hoc selection of teeth for *in-vitro*-detection studies is possible, so that selection of the lesions can be performed more securely and thus a more representative sample size in agreement with epidemiological distributions can be obtained.
